# Addressing the epilepsy treatment gap in low resource settings

**DOI:** 10.1016/j.lanwpc.2024.101081

**Published:** 2024-04-30

**Authors:** The Lancet Regional Health – Western Pacific

According to the 2021 estimates from the Global Burden of Disease Study, neurological disorders are the top contributors to the global disability-adjusted life-years (DALYs) and years of life lost (YLLs). Epilepsy is a brain condition that causes recurrent seizures, with brain injuries and infections being common causes. As one of the most common neurological disorders affecting 51 million people, it was one of the ten conditions with the highest age-standardised DALYs in the general population and the third leading cause of DALYs in older children and adolescents aged 5–19 years. Apart from the disease burden, one of the biggest challenges in epilepsy management is the enormous treatment gap in low-income and middle-income countries (LMICs). As reported by International League Against Epilepsy, the epilepsy treatment gap ranged from fairly low in high-income countries, such as 5·6% in Norway, to 100% in low-income African countries such as Togo and Uganda, showing a significantly higher epilepsy treatment gap in Southern Africa than in other low-resource settings. Treatment gap refers to inappropriate, ineffective treatment and supportive care, which stems from and affects every step of epilepsy management—understanding of the disease, diagnosis, treatment, and lifelong support.

Classification of epilepsy is complicated and relies largely on detailed medical history and diagnostic techniques. Accurate diagnosis of epilepsy can be more difficult in low-resource settings due to the lack of reliable medical records and limited diagnostic expertise and testing tools (eg, electroencephalography, neuroimaging, and genetic sequencing). Misdiagnosis will further lead to inappropriate treatment decisions, as the efficacy and safety of antiseizure medications depends on an individual’s epilepsy or seizure type and general health. Comorbidity with physical, psychological, and cognitive conditions, particularly depression and anxiety, can further complicate effective management. People with epilepsy are at higher risks of premature death and may experience socioeconomic hardship over the course of their life. Social stigma can isolate them and reduce their chances of attending schools, being employed, or getting married. As such, the high medical cost of epilepsy goes beyond direct medical costs; out-of-pocket expenses and loss of work productivity pose a substantial financial burden for individuals and their families.

The effective usage of antiseizure medication can be also impaired by the insufficient epilepsy research and guidelines produced in low-resource settings. Despite recent advances in the high efficacy of antiseizure medication, these clinical trials and real-world data mostly originate from high-income settings and might not be generalisable to LMICs considering cultural and ethnic differences. Another obstacle to appropriate antiseizure medication in LMICs is their limited supply and affordability issues. For example, Laos had no newer (second and third generation) antiseizure medications; the availability of the lowest-priced generic antiseizure medications in Ethiopia was only 52%, and all available medications were unaffordable. For patients with drug-resistant focal epilepsy, surgery is cost-effective but underutilised in LMICs due to the deficit in epilepsy surgery centres and neurosurgeons. Neuromodulation and dietary therapies can be treatment options for those who are not suitable for surgery, and they are showing promising results in high-income countries; however, more evidence is needed for their applicability and guidelines for use in low-resource settings. Beyond health care, innovative strategies are needed to provide longitudinal support in societal involvement for people living with epilepsy.

To address the treatment gap of epilepsy, international and national efforts are on the way. In May 2022, WHO launched the Intersectoral Global Action Plan on Epilepsy and Other Neurological Disorders 2022–2031 (IGAP), whereby strengthening the public health approach to epilepsy is one of the five strategic objectives. IGAP is receiving global recognition and support—for example, its member states in Southeast Asia are urged to achieve IGAP's strategic targets by embracing novel approaches and strengthening existing policies and practices. However, there is concern that current progress is still insufficient and attainment of 2031 IGAP targets is at risk. It could be too early to say, or it may reflect the fact that in LMICs where the capacity of health-care systems is insufficient, research funding and medical resources are allocated to more life-threatening conditions such as infectious diseases and cancers, and there may be low priority assigned to epilepsy.

Therefore, simple, cost-effective ways are more feasible to reduce treatment gaps in LMICs, such as to dispel stigma through public education, develop inexpensive prevention interventions, ensure the continuous supply of good quality antiseizure medications, and integrate epilepsy management into community-based or primary health-care centre-based systems. The latter has been conducted in several LMICs and proven to be feasible and effective. For instance, two community strategies involving domestic health visitors for epilepsy significantly reduced the treatment gap by 5% in Laos and 26% in Cambodia in 1 year, indicating that community strategies are a cost-effective way to address the issue of low identification and diagnosis rates and poor adherence to treatment in low-resource settings. A 2-year training programme for community health workers and general practitioners in Myanmar has proven effective in improving their Knowledge-Attitudes-Practices about epilepsy and reducing the treatment gap. Therefore, training and educating less specialised health-care workers can address the long-term shortage of neurologist specialists in the short term. Nevertheless, primary health-care provider-based diagnosis and management needs to be strengthened, as suggested by a study showing insufficient competence in diagnosing epilepsy of village clinicians in rural China.

Addressing the epilepsy treatment gap in low-resource settings cannot be further delayed. Urgent actions are needed to increase public awareness and to further research on feasible and cost-effective approaches to improve the management of epilepsy in LMICs.

